# Daytime Idle Energy Reduction in Radiotherapy Simulation Computed Tomography: A Prospective Workflow‐Based Evaluation

**DOI:** 10.1002/jmrs.70133

**Published:** 2026-11-02

**Authors:** Kouta Hirotaki, Yoshiaki Honda, Masashi Ito

**Affiliations:** ^1^ Department of Radiological Technology National Cancer Center Hospital East Kashiwa, Chiba Japan; ^2^ Department of Radiology Saiseikai Niigata Kenoh Kikan Hospital Niigata Japan

**Keywords:** computed tomography, energy conservation, power consumption, radiotherapy planning, sustainability

## Abstract

**Introduction:**

Previous computed tomography (CT) energy‐saving studies have primarily focused on after‐hours shutdown or idle energy use in diagnostic CT. Radiotherapy simulation, however, includes preparation and immobilisation steps that may prolong daytime non‐acquisition periods. This study quantified daytime energy use in radiotherapy simulation CT and evaluated the contribution of power‐saving operation.

**Methods:**

In this prospective single‐centre study, electrical power consumption was directly measured using a three‐phase power logger during five baseline and five power‐saving weekdays (08:00–18:00). Power measurements were integrated with patient in‐room intervals and simulation procedure categories. Staff perceptions of workflow impact were assessed using an anonymous questionnaire, and carbon implications were estimated from the measured energy reduction.

**Results:**

Mean directly measured clinical‐hour energy consumption decreased from 38.7 to 36.5 kWh/day with power‐saving operation, a 5.8% reduction. Non‐acquisition time during patient presence varied markedly by procedure, from 56.5% for respiratory‐gated four‐dimensional CT to 97.1% for head‐and‐neck simulation. Power‐saving operation shifted daytime power use towards a lower‐power state, corresponding to an estimated reduction of 0.84–1.01 kg‐CO_2_ per clinical day across the emission‐factor sensitivity range. Twenty of 21 staff (95.2%) reported no impact on patient throughput.

**Conclusion:**

Power‐saving operation reduced daytime energy consumption without meaningful workflow disruption. Radiotherapy simulation CT has prolonged, procedure‐dependent idle periods arising from radiotherapy‐specific preparation workflows, highlighting a practical and previously undercharacterised opportunity for energy reduction.

## Graphical abstract

Radiotherapy simulation CT includes prolonged, procedure‐dependent non‐acquisition periods associated with treatment preparation and immobilisation. Built‐in power‐saving operation shifted idle power demand to a lower‐power state and reduced daytime energy consumption by 5.8% without meaningful workflow disruption. Generative AI was used to create some illustrative elements in the graphical abstract.

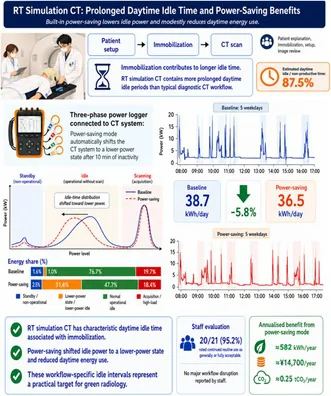

## 1 Introduction

The healthcare sector is increasingly recognised as a substantial contributor to greenhouse gas emissions, representing approximately 4.4% of global emissions. Accordingly, improving the environmental sustainability of healthcare delivery has become a major priority for modern health systems [1, 2, 3]. Diagnostic imaging plays a critical role in healthcare sustainability because imaging services depend on energy‐intensive equipment and digital infrastructure. Advanced imaging modalities such as computed tomography (CT) and magnetic resonance imaging contribute disproportionately to this environmental burden [4, 5, 6].

Among imaging modalities, CT warrants particular attention owing to its substantial electricity demand during both active scanning and non‐acquisition operating states. Previous studies have shown that a considerable proportion of CT‐related energy consumption occurs while the system remains powered without actively acquiring images [7, 8, 9]. Energy‐saving strategies have consequently focused mainly on overall system consumption, after‐hours shutdown, or low‐power operation during non‐operational periods [8, 9, 10]. Energy use during routine clinical hours, particularly within radiotherapy‐specific workflows, has been less well characterised.

Radiotherapy simulation CT represents a distinct clinical application in which image acquisition is embedded within a treatment‐planning workflow requiring reproducible patient positioning, immobilisation‐device preparation, and other procedure‐specific preparation [11, 12, 13, 14, 15]. These processes can create prolonged non‐acquisition intervals between scans, and their duration may vary substantially by simulation procedure. However, the energy implications of these radiotherapy‐specific workflow characteristics remain poorly characterised, as does the extent to which power‐saving operation can exploit such intervals without disrupting routine clinical workflow.

Therefore, this study aimed to directly quantify daytime energy consumption in dedicated radiotherapy simulation CT under routine clinical workflow conditions and to evaluate the contribution of power‐saving operation using direct three‐phase electrical power measurements.

## 2 Materials and Methods

### 2.1 Study Design

This prospective single‐centre measurement study was designed to characterise the energy consumption of a clinical CT system used for radiotherapy simulation and to evaluate the effect of a built‐in power‐saving mode under routine clinical operating conditions. A predefined study protocol was established before data acquisition. Measurements were performed prospectively in a real‐world clinical setting without altering routine clinical workflow, except for implementation of the predefined power‐saving intervention. The primary endpoint was daily energy consumption during the predefined clinical‐hour period. Additional analyses evaluated power‐defined operational‐state occupancy, procedure‐specific patient in‐room and non‐acquisition intervals, energy consumption per simulation, staff‐reported workflow impact and acceptability, and estimated economic and carbon dioxide (CO_2_) implications. A workload‐standardised reconstruction was also performed to assess the potential influence of differences in workload and procedural composition between the study phases. The Institutional Review Board of the National Cancer Center Japan determined that formal ethical review was not required for this study, and written documentation of this determination was obtained (Reference No. 6000‐137).

### 2.2 CT System and Clinical Workflow

A dedicated CT system (Aquilion ONE, Canon Medical Systems, Otawara, Japan), installed in 2022, was used exclusively for radiotherapy simulation in the radiation oncology department. The system was operated under routine clinical conditions, with scheduled working hours from 08:00 to 17:15; however, image review and prolonged simulation procedures extended the effective operational period to approximately 08:00–18:00. A lunch break was routinely scheduled between 12:00 and 13:00, during which no patient imaging was performed unless clinically required. During the primary comparison periods, 55 simulations were performed during baseline operation and 52 during power‐saving operation, corresponding to 11.0 ± 1.9 and 10.4 ± 1.1 simulations per day, respectively. Major simulation workflows included respiratory‐gated/four‐dimensional CT, head‐and‐neck simulation requiring immobilisation‐device preparation, deep‐inspiration breath‐hold simulation, and brain, pelvic, thoracoabdominal, and palliative simulations. Radiotherapy simulation workflows included patient explanation, site‐specific treatment positioning, immobilisation‐device preparation, contrast preparation when required, respiratory management, and post‐acquisition image review for treatment‐planning suitability. Procedure duration varied substantially according to the complexity of these workflow requirements.

### 2.3 Power Measurement System

Electrical power consumption was directly measured using a portable three‐phase power logger (PW3360‐11, HIOKI, Nagano, Japan) equipped with three clamp‐on current sensors (model 9661, HIOKI), enabling simultaneous measurement of all three phases. The measurement system was installed by a qualified electrical engineer at the distribution board supplying the CT system through a dedicated power line, allowing electrical consumption attributable to the CT system to be measured directly. The CT system was supplied by three‐phase 415‐V electricity. Phase‐specific voltage and current were measured, and active power was calculated internally by the logger. Active power was continuously recorded at 5‐s intervals and stored digitally for subsequent analysis under routine clinical operating conditions. Recorded power and workflow data were analysed by the study investigators.

### 2.4 Study Protocol and Operational Phases

The study was conducted prospectively under routine clinical operating conditions using two sequential measurement phases. The baseline phase was conducted over five weekdays from 1 to 5 June 2026 under standard operating conditions. The power‐saving phase was conducted over five weekdays from 8 to 12 June 2026 with the predefined power‐saving mode enabled. The primary comparison was restricted to the predefined clinical‐hour period from 08:00 to 18:00 on each study day. Except for activation of the predefined power‐saving setting, no modifications were made to the routine radiotherapy simulation workflow during either phase. The phase sequence and primary measurement periods were determined prospectively before data acquisition. In addition to the primary five‐weekday comparison, power monitoring under the power‐saving condition was extended through 13 July 2026. The available measurements from this extended period were analysed descriptively using the same operational‐state definitions to assess whether the operational‐state pattern observed during the primary power‐saving phase was maintained over a longer period.

### 2.5 Power‐Saving Mode Configuration

The CT system was an Aquilion ONE (TSX‐306A; software version V10.12, release SP0006J; Base V15.1, release SP0204; Canon Medical Systems). During the power‐saving phase, the manufacturer‐integrated Power Save Mode was configured to activate after 10 min of inactivity; the function was disabled during the baseline phase. No other CT system settings were modified between phases. According to the manufacturer's operating instructions, Power Save Mode switches off selected components, including the gantry/couch servo system, the main circuit of the high‐voltage generator, and the station and console monitors, with normal operation resumed by patient registration or gantry operation [16]. In routine clinical use, recovery was observed to occur within a few seconds without requiring system restart.

### 2.6 Workflow‐Integrated Profile Analysis

Patient room‐entry and room‐exit times and simulation procedure categories were obtained from the radiology information system (RIS), with relevant workflow events recorded contemporaneously by radiological technologists. Scanner internal logs were not used. Workflow data were synchronised with the 5‐s active‐power measurements. Patient in‐room duration was defined from RIS‐recorded room entry to room exit. Acquisition‐related time was defined as the cumulative duration of measurements within the in‐room interval meeting the acquisition/high‐load threshold, and non‐acquisition time as the remaining in‐room duration. Time to first acquisition‐related activity was measured from room entry to the first acquisition/high‐load event. Procedure‐specific analyses were performed for the major radiotherapy simulation categories. For visualisation, 5‐s power data were additionally aggregated into 1‐min mean profiles. Operational states were classified using thresholds derived from local minima in the observed 5‐s power distributions: standby/non‐operational (< 1.80 kW), lower‐power (1.80 to < 3.125 kW), normal operational idle (3.125 to < 5.35 kW), and acquisition/high‐load (≥ 5.35 kW). These categories were based on measured power behaviour and interpreted together with RIS and workflow information.

### 2.7 Staff Workflow Assessment

Following implementation of the power‐saving mode, an anonymous online questionnaire was administered via Google Forms to 21 radiological technologists involved in radiotherapy CT simulation. All 21 invited staff members responded (response rate, 100%). No names, email addresses, or other personally identifiable information were collected. Six items assessed workflow delay, patient throughput, additional operational burden, technical problems, immediate scanner availability, and acceptability for routine use. Questions on specific workflow or technical concerns used yes/no responses, whereas routine‐use acceptability was assessed on a five‐point scale. The complete English translation of the questionnaire is provided in Appendix S1.

### 2.8 Statistical, Economic, and Carbon Analyses

Daily clinical‐hour energy consumption was summarised as mean ± standard deviation, and energy consumption per simulation was also calculated. Given the five weekdays available in each phase, the primary analysis focused on descriptive effect estimates and workload‐standardised reconstruction rather than formal inferential testing. To assess the influence of differences in workload and procedural composition, a workload‐standardised reconstruction was performed using the directly measured 5‐s power data under a common pooled workload. This analysis was treated as a reconstructed sensitivity analysis and reported separately from the directly measured comparison. Economic and carbon implications were derived from the directly measured mean daytime electricity reduction. Electricity‐cost savings used the institutional rate of 25.2 JPY/kWh. CO_2_ reductions were estimated using the FY2024 adjusted electricity emission factor of 0.421 kg‐CO_2_/kWh reported by Tokyo Electric Power Company Energy Partner. A sensitivity analysis used the corresponding published FY2021–FY2024 factors (0.376–0.451 kg‐CO_2_/kWh) to account for temporal variation in grid carbon intensity [17]. Annualised estimates assumed 260 clinical days. These estimates did not include potential savings from after‐hours or weekend shutdown.

## 3 Results

### 3.1 Daytime Energy Consumption

During the primary comparison period, 55 simulations were performed during the baseline phase and 52 during the power‐saving phase. Mean patient‐present time was 199.8 ± 22.0 and 189.0 ± 55.3 min/day, respectively, while acquisition‐related time was 43.0 ± 11.3 and 39.5 ± 17.3 min/day (Table 1). Mean directly measured clinical‐hour energy consumption decreased from 38.71 ± 0.97 kWh/day during baseline operation to 36.47 ± 2.52 kWh/day during power‐saving operation (Figure 1), corresponding to an absolute reduction of 2.24 kWh/day and a relative reduction of 5.78%. Energy consumption per simulation was 3.60 ± 0.57 and 3.53 ± 0.30 kWh, respectively (Table 1). After standardising patient‐present time and acquisition‐related activity to a common pooled workload, reconstructed clinical‐hour energy consumption was 39.82 kWh/day for the baseline operational‐state structure and 37.52 kWh/day for the power‐saving structure, corresponding to a 5.77% reduction. Results across alternative workload‐standardisation scenarios are provided in Table S1.

**TABLE 1:** Clinical workload and primary energy outcomes during baseline and power‐saving operation.

Metric	Baseline	Power‐saving	Difference
*Clinical workload*			
Simulations, *n*	55	52	−3
Patients/day	11.0 ± 1.9	10.4 ± 1.1	−0.6 (−5.5%)
Patient‐present time/day, min	199.8 ± 22.0	189.0 ± 55.3	−10.8 (−5.4%)
Acquisition‐related time/day, min	43.0 ± 11.3	39.5 ± 17.3	−3.6 (−8.3%)
*Directly measured energy*			
Clinical‐hour energy/day, kWh	38.71 ± 0.97	36.47 ± 2.52	−2.24 (−5.78%)
Clinical‐hour energy/simulation, kWh	3.60 ± 0.57	3.53 ± 0.30	−0.07 (−1.90%)
*Operational‐state occupancy during clinical hours*			
Standby/non‐operational, %	7.12	5.93	−1.19 pp
Lower‐power state, %	1.22	39.13	+37.91 pp
Normal operational idle, %	84.49	48.36	−36.13 pp
Acquisition/high‐load, %	7.17	6.58	−0.59 pp
*Workload‐standardised reconstruction*			
Reconstructed clinical‐hour energy/day, kWh	39.82	37.52	−2.30 (−5.77%)

*Note:* Daily values are mean ± SD unless otherwise indicated. Operational‐state occupancy represents the pooled proportion of 5‐s measurements during clinical hours (08:00–18:00). Workload‐standardised values are reconstructed estimates under a common workload of 194.4 min/day of patient‐present time and 41.23 min/day of acquisition‐related activity.

Abbreviation: pp, percentage points.

**FIGURE 1:**
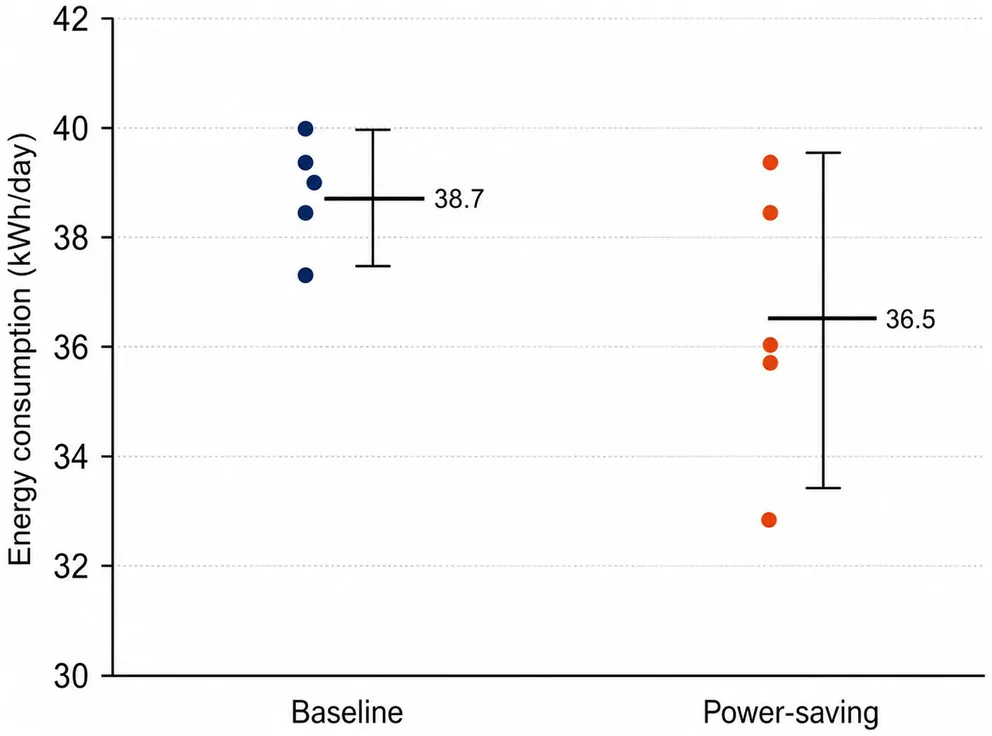
Daytime energy consumption during the baseline and power‐saving phases. Daily clinical‐hour energy consumption (08:00–18:00) is shown for each of the five weekdays in the baseline and power‐saving phases. Individual points represent daily measured values. Horizontal bars indicate the mean, and error bars indicate the standard deviation. Mean energy consumption decreased from 38.7 kWh/day during baseline operation to 36.5 kWh/day during power‐saving operation.

### 3.2 Shift in Daytime Operational‐State Distribution

The distribution of operational states during clinical hours differed markedly between the two phases. The lower‐power state accounted for 1.22% of measured time during baseline operation and 39.13% during power‐saving operation, whereas normal operational idle decreased from 84.49% to 48.36% (Table 1). The proportion of time classified as acquisition/high‐load was similar between phases (7.17% vs. 6.58%), as was standby/non‐operational time (7.12% vs. 5.93%). Corresponding changes in the contribution of each operational state to clinical‐hour energy consumption are shown in Figure S1.

In the extended monitoring dataset, lower‐power‐state occupancy during weekday clinical hours was 39.50%, compared with 39.13% during the primary 5‐day power‐saving period (Table S3).

### 3.3 Power Distribution During Clinical Operation

The distribution of 5‐s active‐power measurements differed between the baseline and power‐saving phases (Figure 2). During baseline operation, the dominant clinical‐hour peak was located within the normal operational idle range, whereas power‐saving operation produced an additional prominent peak within the lower‐power range and reduced the relative concentration of measurements in the normal operational idle range. In contrast, the distributions within the acquisition/high‐load range broadly overlapped between phases. Standby/non‐operational measurements were also present in both phases without a marked phase‐dependent shift.

**FIGURE 2:**
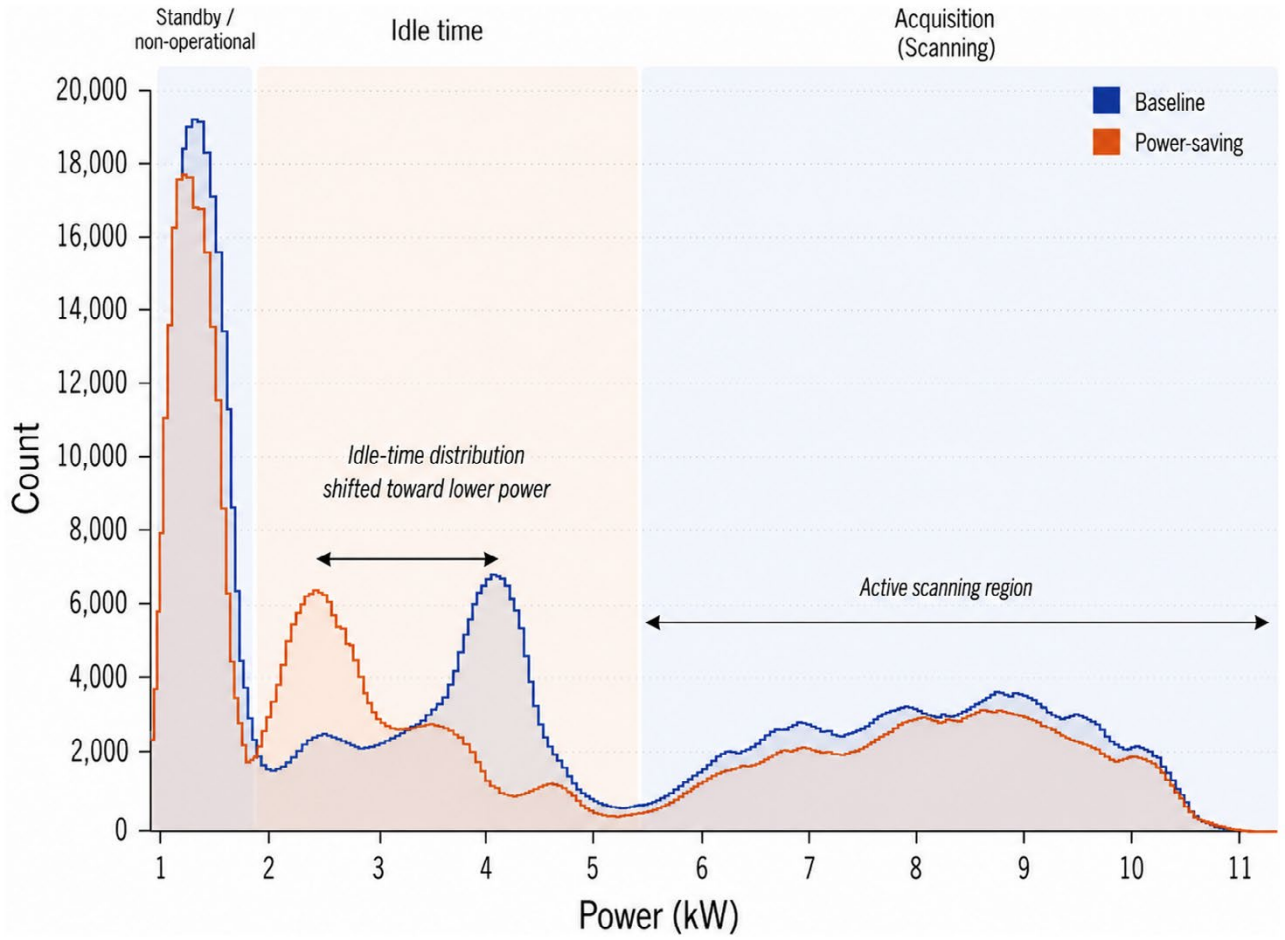
Distribution of active power measurements during the baseline and power‐saving phases. Distribution of 5‐s active‐power measurements recorded during clinical hours (08:00–18:00) in the baseline and power‐saving phases. Power‐saving operation shifted a substantial proportion of measurements from the normal operational idle range towards the lower‐power range, whereas the distributions within the acquisition/high‐load range were broadly similar between phases. The shaded regions indicate the operational ranges used for interpretation: Standby/non‐operational (< 1.80 kW), idle operation (1.80 to < 5.35 kW), and acquisition/high‐load activity (≥ 5.35 kW). Within the idle range, the lower‐power state was defined as 1.80 to < 3.125 kW and normal operational idle as 3.125 to < 5.35 kW.

### 3.4 Procedure‐Specific Radiotherapy Simulation Workflow

Procedure‐specific analysis demonstrated substantial variation in the temporal composition of radiotherapy simulation workflows. Respiratory‐gated/4DCT simulations had a median in‐room duration of 26.0 min, with 10.0 min of acquisition‐related activity and a median non‐acquisition proportion of 56.5%. In contrast, head‐and‐neck simulations had a similar median in‐room duration of 27.5 min but only 0.7 min of acquisition‐related activity, with 97.1% of the in‐room interval classified as non‐acquisition time. High non‐acquisition proportions were also observed in specialised simulation workflows. The two DIBH simulations had a median non‐acquisition proportion of 95.9%, while the single TBI and CSI simulations showed non‐acquisition proportions of 97.5% and 97.3%, respectively. The corresponding time to first acquisition‐related activity was 8.3 min for DIBH, 28.2 min for TBI, and 22.0 min for CSI (Table 2). The 10‐day workflow‐integrated power profiles showed corresponding procedure‐dependent temporal patterns (Figure 3). Respiratory‐gated/4DCT procedures showed broader acquisition‐related power activity, whereas head‐and‐neck and other preparation‐intensive simulations showed longer intervals between patient room entry and acquisition‐related activity.

**TABLE 2:** Procedure‐specific radiotherapy simulation workflow characteristics.

Procedure	Baseline, n	Power‐saving, n	In‐room duration, min	Time to first acquisition, min	Acquisition‐related time, min	Non‐acquisition time, %
Respiratory gated (4DCT)	13	9	26.0 (23.0–31.8)	7.7 (6.9–11.0)	10.0 (7.3–15.2)	56.5 (50.4–69.2)
Head and neck	9	5	27.5 (13.5–30.0)	17.4 (9.5–21.1)	0.7 (0.6–0.8)	97.1 (95.3–97.8)
DIBH	2	0	26.5 (26.2–26.8)	8.3 (7.7–9.0)	1.1 (1.0–1.1)	95.9 (95.7–96.1)
TBI	0	1	44.0	28.2	1.1	97.5
CSI	0	1	31.0	22.0	0.8	97.3
Brain	3	1	15.5 (14.2–17.2)	12.1 (10.0–13.9)	0.5 (0.5–0.5)	96.5 (96.2–96.9)
Thoracoabdominal (non‐4D)	5	3	19.0 (14.0–27.5)	9.0 (8.2–11.4)	0.8 (0.6–1.7)	94.1 (90.5–96.5)
Pelvic	10	16	10.5 (8.2–13.0)	5.5 (4.7–7.1)	0.7 (0.5–0.7)	94.3 (93.0–95.8)
Palliative	9	8	9.0 (6.0–14.0)	6.1 (4.1–8.2)	0.6 (0.5–0.7)	93.8 (91.7–95.6)

*Note:* Values are median (interquartile range) unless only one procedure was available. Time to first acquisition was measured from radiology information system room‐entry time to the first power‐defined acquisition/high‐load event. Acquisition‐related time represents cumulative time at ≥ 5.35 kW within the patient in‐room interval and is a power‐defined metric, not scanner‐log or DICOM series duration. Non‐acquisition time was calculated as the proportion of the in‐room interval below 5.35 kW.

Abbreviations: 4DCT, four‐dimensional computed tomography; CSI, craniospinal irradiation; DIBH, deep‐inspiration breath hold; TBI, total body irradiation.

**FIGURE 3:**
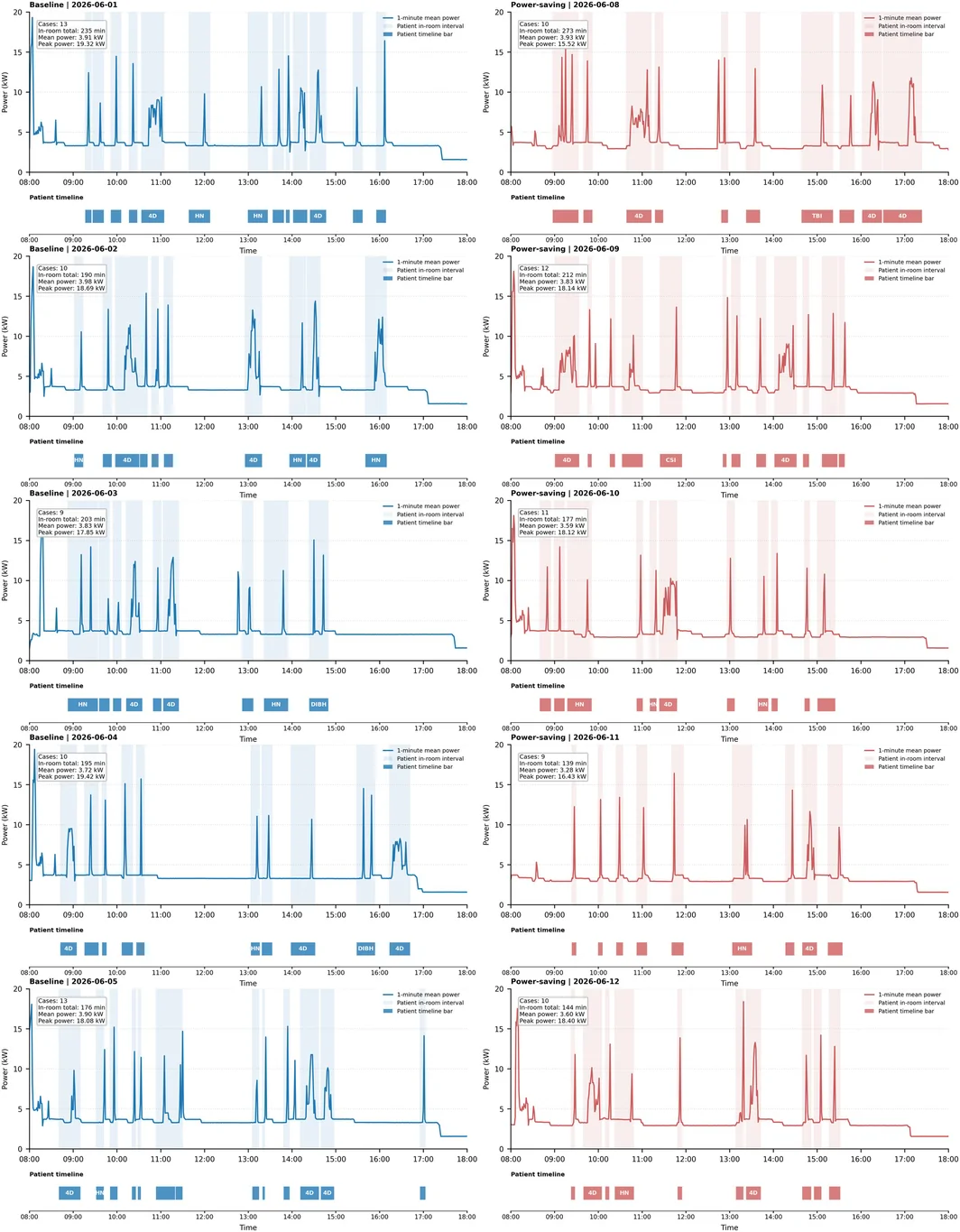
Workflow‐integrated daytime power profiles during the primary 10‐day study period. One‐minute mean power profiles are shown for each of the five baseline weekdays (left) and five power‐saving weekdays (right) during clinical hours from 08:00 to 18:00. Shaded vertical regions indicate patient in‐room intervals derived from the radiology information system, and the horizontal patient‐timeline bars indicate individual simulation procedures. Procedure labels identify selected radiotherapy simulation categories, including respiratory‐gated/four‐dimensional CT (4D), head and neck (HN), deep‐inspiration breath hold (DIBH), total body irradiation (TBI), and craniospinal irradiation (CSI). The panels illustrate the temporal relationship between scanner power consumption, patient presence, and procedure‐specific radiotherapy simulation workflows.

### 3.5 Staff Workflow Assessment

All 21 radiological technologists completed the staff questionnaire. Twenty respondents (95.2%) reported no delay in patient throughput or examination progress, and all 21 (100%) reported no technical problems related to the power‐saving mode. Routine use was rated as generally or fully acceptable by 20 respondents (95.2%), including 18 (85.7%) who rated it fully acceptable. Additional findings regarding workflow delay, operational burden, and immediate scanner availability are summarised in Table 3.

**TABLE 3:** Staff evaluation of the Power Save Mode in routine radiotherapy simulation CT.

Survey domain	Yes, *n* (%)	No, *n* (%)	Summary
Workflow delay during recovery	1 (4.8%)	20 (95.2%)	20/21 (95.2%)
Delay in patient throughput/examination progress	1 (4.8%)	20 (95.2%)	20/21 (95.2%)
Additional operational burden	3 (14.3%)	18 (85.7%)	18/21 (85.7%)
Technical problems related to power‐saving mode	0 (0.0%)	21 (100.0%)	21/21 (100%)
Impaired immediate scanner availability	2 (9.5%)	19 (90.5%)	19/21 (90.5%)
Acceptability for routine use			
5—Fully acceptable			18/21 (85.7%)
4—Generally acceptable with only minor impact			2/21 (9.5%)
3—Minor impact but not actively acceptable			1/21 (4.8%)
2—Not very acceptable			0/21 (0.0%)
1—Not acceptable at all			0/21 (0.0%)
Generally or fully acceptable (scores 4–5)			20/21 (95.2%)

*Note:* The anonymous questionnaire was administered via Google Forms to 21 staff members involved in radiotherapy CT simulation. For Questions 1–5, a “Yes” response indicated the presence of the specified workflow or technical concern. Routine‐use acceptability was rated on a 5‐point scale, with scores of 4 or 5 considered generally or fully acceptable. The complete English translation of the questionnaire is provided in Appendix S1.

### 3.6 Economic and Carbon Implications

The directly measured mean daytime electricity reduction of 2.24 kWh/day corresponded to an estimated electricity‐cost saving of 56.4 JPY per clinical day and 14,663 JPY over 260 clinical days. The estimated CO_2_ reduction was 0.942 kg‐CO_2_ per clinical day using the FY2024 reference emission factor, with a sensitivity range of 0.841–1.009 kg‐CO_2_/day across the published FY2021–FY2024 emission factors. Annualised over 260 clinical days, these values corresponded to 582 kWh of electricity and 218.8–262.4 kg‐CO_2_ (Table 4).

**TABLE 4:** Economic and carbon implications of the directly measured daytime energy reduction.

Outcome	Assumption	Per clinical day	260 clinical days
Electricity saving	Directly measured	2.24 kWh	582 kWh
Electricity‐cost saving	25.2 JPY/kWh	56.4 JPY	14,663 JPY
Reference estimate	0.421 kg‐CO_2_/kWh	0.942 kg‐CO_2_	245.0 kg‐CO_2_
Sensitivity range	0.376–0.451 kg‐CO_2_/kWh	0.841–1.009 kg‐CO_2_	218.8–262.4 kg‐CO_2_

*Note:* Economic and CO_2_ values are derived estimates based on the directly measured mean daytime electricity saving of 2.24 kWh/day. Annualised values assume 260 clinical days and do not represent the previously modelled 24‐h/7‐day shutdown scenario. The electricity price was obtained from institutional data applicable to the study period. The reference CO_2_ factor was 0.421 kg‐CO_2_/kWh; sensitivity analysis used 0.376–0.451 kg‐CO_2_/kWh to reflect published regional temporal variation.

Abbreviation: CO_2_, carbon dioxide.

## 4 Discussion

This prospective direct‐measurement study demonstrated a 5.8% reduction in daytime energy consumption after activation of a built‐in power‐saving mode in a dedicated radiotherapy simulation CT system. A similar reduction was obtained after workload standardisation. Power‐saving operation shifted the idle‐period power profile towards a lower‐power range, providing energy savings during periods in which the scanner remained clinically available but was not actively acquiring images.

Previous studies have established non‐acquisition operation as an important target for reducing CT energy consumption, although the strategies and clinical settings have varied. Heye et al. demonstrated that substantial CT energy consumption occurs outside active image acquisition [7]. Brown et al. and Marks et al. [8, 9] showed the benefit of switching scanners off during prolonged periods when imaging was not required. More directly comparable with the present study, Hehenkamp et al. [10] evaluated a rapid‐reactivation power‐save mode during short inter‐examination idle periods in diagnostic CT and reported reductions in both idle‐state and total energy consumption. Stortz et al. [18] subsequently modelled the use of low‐power modes according to CT utilisation and suggested greater potential savings in scanners with light or intermittent use. Radiology‐department‐level modelling has likewise identified scheduled power‐management strategies as a potential source of energy reduction [19]. The present study extends this concept to daytime radiotherapy simulation, focusing specifically on idle periods generated within the clinical workflow itself and directly measuring the energy reduction achievable during these intervals.

Radiotherapy simulation differs fundamentally from routine diagnostic CT because the CT examination also establishes the treatment position. The position defined at simulation must be comfortable, reproducible, and sufficiently stable to be maintained during subsequent treatment sessions, often over several weeks. Accordingly, substantial clinical activity may occur in the CT room before image acquisition, including patient instruction, confirmation of treatment and preparation requirements, positioning and adjustment on the treatment couch, preparation of patient‐specific immobilisation devices, determination of the treatment reference position and scan range, and respiratory preparation when required. These intervals are therefore clinically necessary components of radiotherapy preparation rather than unused downtime. Their relatively long duration creates a potentially favourable opportunity for power‐saving operation while treatment preparation is being performed.

The procedure‐specific findings further illustrate the heterogeneity of these workflows. Respiratory‐gated/4DCT and head‐and‐neck simulations had similar in‐room durations, but acquisition‐related activity occupied 10.0 min in respiratory‐gated/4DCT compared with only 0.7 min in head‐and‐neck simulation, resulting in markedly different non‐acquisition proportions. Respiratory‐gated/4DCT requires repeated or prolonged imaging‐related activity, whereas head‐and‐neck simulation commonly involves creation of a customised head support and thermoplastic mask before imaging. Other specialised procedures generate different patterns: DIBH requires confirmation of reproducible deep inspiration before acquisition, whereas TBI and CSI may involve extensive patient‐specific immobilisation and positioning. In paediatric CSI, obtaining a stable treatment position and sufficient cooperation may add further preparation time. Thus, non‐acquisition time in radiotherapy simulation is strongly procedure dependent and reflects the clinical requirements of treatment preparation rather than a uniform period of scanner inactivity.

The practical value of this approach lies in its ability to reduce power demand without materially changing the clinical workflow. Recovery from the lower‐power condition occurred within a few seconds and required only simple user interaction, without additional hardware, imaging‐protocol modification, or substantive staff training. Consistent with this experience, 95.2% of staff reported no effect on patient throughput, all respondents reported no technical problems, and 95.2% considered routine use generally or fully acceptable. Power‐saving operation may therefore be particularly applicable to scanners that must remain available throughout the working day but are used intermittently. This includes lower‐volume radiotherapy simulation units and CT systems maintained in a ready state despite relatively infrequent examinations. Similar principles may apply to low‐volume or on‐call diagnostic CT systems. Conversely, the opportunity for energy reduction is likely to be smaller in high‐volume diagnostic CT units in which examinations are performed almost continuously and opportunities to enter a lower‐power state are limited. This utilisation‐dependent relationship is consistent with previous modelling of low‐power CT operation [18]. Extended monitoring in the present study showed a lower‐power‐state occupancy similar to that observed during the primary intervention period, supporting the persistence of the operational pattern beyond the initial 5‐day comparison.

From an environmental perspective, even a relatively simple daytime intervention can contribute additional energy savings alongside established shutdown practices. In the present study, the measured reduction corresponds to approximately 582 kWh annually under the assumed 260 clinical days, with the estimated CO_2_ benefit remaining within a similar range across the regional emission factors examined. Previous studies have reported larger annual savings when shutdown or low‐power strategies were applied over prolonged nights and weekends [8, 9, 18], which is expected given the substantially longer intervention windows. The contribution of daytime power‐saving is therefore complementary: it addresses shorter periods during which complete shutdown is impractical because the scanner must remain immediately available. Because the intervention requires no additional equipment and minimal operational change, implementation across multiple suitable scanners could provide an additional practical component of broader sustainability initiatives in radiotherapy and medical imaging [20].

Several limitations should be considered. First, this was a single‐centre, single‐scanner study and may therefore be subject to institution‐ and system‐specific bias. Second, the primary comparison was limited to five weekdays per phase, and patient volume and procedural composition were not controlled between the sequential study periods. Third, operational states were defined from measured power profiles rather than scanner‐internal logs. Fourth, staff workflow was assessed subjectively using a questionnaire. Fifth, the annualised economic and carbon estimates depend on the assumptions used for their calculation.

## 5 Conclusion

Power‐saving operation reduced daytime energy consumption in radiotherapy simulation CT without meaningful disruption to clinical workflow. Radiotherapy‐specific workflows, including patient‐specific immobilisation and treatment‐position preparation, can prolong non‐acquisition periods and may therefore provide favourable opportunities for power‐saving operation. Effective use of such built‐in power‐saving functions, tailored to the characteristics of radiotherapy simulation workflows, may offer a practical approach to advancing greener and more sustainable radiotherapy practice.

## Author Contributions

Kouta Hirotaki: Conceptualization, Methodology, Investigation, Formal analysis, Visualisation, Writing – original draft, Writing – review and editing. Yoshiaki Honda: Methodology, Writing – review and editing. Masashi Ito: Supervision, Project administration, Writing – review and editing. All authors approved the final version of the manuscript.

## Funding

English editing and publication‐related expenses for this study were supported by collaborative research funds from Hitachi High‐Tech Corporation. The funder had no role in the study design, data collection, data analysis, data interpretation, manuscript preparation, or decision to submit the manuscript for publication.

## Ethics Statement

The Institutional Review Board of the National Cancer Center Japan determined that formal ethical review was not required for this study, and written documentation of this determination was obtained (No. 6000‐137).

## Conflicts of Interest

The authors declare that English editing and publication‐related expenses were supported by collaborative research funds from Hitachi High‐Tech Corporation. Hitachi High‐Tech Corporation had no involvement in the study design, data collection, data analysis, data interpretation, manuscript preparation, or decision to submit the manuscript. The authors declare no other conflicts of interest.

## Supporting information


**Appendix S1:** English translation of the staff questionnaire.

**Table S1:** Workload‐standardisation sensitivity analyses.

**Table S2:** Contextual comparison with published CT energy‐use studies.

**Table S3:** Extended monitoring during the power‐saving period using the revised operational‐state definitions.

**Figure S1:** Distribution of clinical‐hour time and energy across the four operational power states. The proportions of clinical‐hour time and energy consumption attributable to each operational state are shown for the baseline and power‐saving phases. Operational states were defined from the 5‐s active‐power measurements as standby/non‐operational (< 1.80 kW), lower‐power (1.80 to < 3.125 kW), normal operational idle (3.125 to < 5.35 kW), and acquisition/high‐load (≥ 5.35 kW). Power‐saving operation markedly increased the contribution of the lower‐power state and reduced normal operational idle, whereas acquisition/high‐load occupancy remained similar between phases.

## Data Availability

The data that support the findings of this study are available from the corresponding author upon reasonable request.
