# Supplementary material for: Daytime Idle Energy Reduction in Radiotherapy Simulation Computed Tomography: A Prospective Workflow‐Based Evaluation
Source: J Med Radiat Sci. 2026 Nov 2:10.1002/jmrs.70133. Online ahead of print. doi: 10.1002/jmrs.70133 (PMC13725422; doi:10.1002/jmrs.70133)
Supplement: Supplementary file 1 [file JMRS-9999-0-s001.docx]

Supplementary Figure S1. Distribution of clinical-hour time and energy across the four operational power states.
