# Supplementary material for: Daytime Idle Energy Reduction in Radiotherapy Simulation Computed Tomography: A Prospective Workflow‐Based Evaluation
Source: J Med Radiat Sci. 2026 Nov 2:10.1002/jmrs.70133. Online ahead of print. doi: 10.1002/jmrs.70133 (PMC13725422; doi:10.1002/jmrs.70133)
Supplement: Supplementary file 2 [file JMRS-9999-0-s002.docx]

**Supplementary Appendix 1. English translation of the staff questionnaire**

**Administration note.** The questionnaire was administered anonymously in Japanese using Google Forms. No names, email addresses, or other personally identifiable information were collected. The English wording below is a meaning-preserving translation of the questionnaire used in the study.

**Survey introduction presented to participants.** This questionnaire was designed to evaluate the effect of the power-saving mode introduced for radiotherapy simulation CT on routine clinical workflow. When no operation is performed for a predefined period, the CT system automatically transitions to a lower-power state and returns to normal operation within a few seconds when use is resumed. Staff were asked to respond on the basis of their actual experience using the power-saving mode in routine practice. The estimated completion time was 1–2 minutes.

| **No.** | **Domain** | **Question presented to staff** | **Response options** |
| --- | --- | --- | --- |
| 1 | Workflow delay | When the system returns from power-saving mode to its normal operating state, have you ever perceived a delay in routine CT simulation workflow because of the several-second recovery time? | Yes / No |
| 2 | Patient flow and examination progress | Have you ever felt that the power-saving mode delayed the workflow from patient entry into the CT room to completion of the examination, or the overall progress of CT simulation? | Yes / No |
| 3 | Additional operational burden | Have you ever felt that use of the power-saving mode required additional operations or staff effort beyond routine practice? | Yes / No |
| 4 | Technical problems | Have you experienced any technical problem related to the power-saving mode, such as delayed recovery, temporary inability to operate the system, or the need to restart the scanner? | Yes / No |
| 5 | Immediate scanner availability | Have you ever felt that the CT scanner was not immediately available when needed because the power-saving mode was active? | Yes / No |
| 6 | Acceptability for routine clinical use | To what extent do you consider continued use of the power-saving mode acceptable in routine radiotherapy simulation CT practice? | 1. Completely unacceptable 2. Generally unacceptable 3. Minor impact, but I would not actively accept continued use 4. Minor impact and generally acceptable 5. Fully acceptable |

**Note:** The original questionnaire was presented in Japanese. Questions 1–5 used binary responses; Question 6 used a five-point ordinal response scale. The wording above has been edited only for clarity in English and preserves the intent of the original items.
