# Supplementary material for: Daytime Idle Energy Reduction in Radiotherapy Simulation Computed Tomography: A Prospective Workflow‐Based Evaluation
Source: J Med Radiat Sci. 2026 Nov 2:10.1002/jmrs.70133. Online ahead of print. doi: 10.1002/jmrs.70133 (PMC13725422; doi:10.1002/jmrs.70133)
Supplement: Supplementary file 3 [file JMRS-9999-0-s003.docx]

Supplementary Table S1. Workload-standardisation sensitivity analyses

| **Standardisation scenario** | **Baseline (kWh/day)** | **Power-saving (kWh/day)** | **Difference (kWh/day)** | **Relative difference (%)** |
| --- | --- | --- | --- | --- |
| Pooled 10-day workload | 39.82 | 37.52 | -2.30 | -5.77 |
| Baseline workload | 40.03 | 37.76 | -2.27 | -5.68 |
| Power-saving workload | 39.61 | 37.29 | -2.33 | -5.87 |
| Global non-acquisition standardisation | 38.64 | 36.55 | -2.09 | -5.41 |

Note. The pooled standard workload comprised 194.4 min/day of patient-present time and 41.23 min/day of acquisition-related activity. These are reconstructed sensitivity scenarios rather than additional directly measured intervention outcomes. Across alternative workload assumptions, the estimated reduction remained approximately 5.4–5.9%.
