# Supplementary material for: Daytime Idle Energy Reduction in Radiotherapy Simulation Computed Tomography: A Prospective Workflow‐Based Evaluation
Source: J Med Radiat Sci. 2026 Nov 2:10.1002/jmrs.70133. Online ahead of print. doi: 10.1002/jmrs.70133 (PMC13725422; doi:10.1002/jmrs.70133)
Supplement: Supplementary file 4 [file JMRS-9999-0-s004.docx]

Supplementary Table S2. Contextual comparison with published CT energy-use studies

***A. Idle or nonproductive time***

| **Study (year)** | **Setting / metric** | **Idle or nonproductive time** | **Interpretive note** |
| --- | --- | --- | --- |
| Marks et al. (2025) [9] | Diagnostic CT; estimated weekday daytime idle time | ~60% | Approximate value derived from the reported active-scanning fraction within the weekday clinical window. |
| Hehenkamp et al. (2025) [10] | Diagnostic CT; scanner-on nonproductive time during workdays | 66.1% | Represents nonproductive scanner-on time; operational definitions differed from the present study. |
| Present study (2026) | Radiotherapy simulation CT; weekday clinical hours during power-saving operation | 87.5% | Lower-power state plus normal operational idle (39.13% + 48.36%). |

Note. Idle-state definitions and observation windows differed among studies. Values are therefore intended to provide contextual, rather than directly equivalent, comparison of daytime non-acquisition opportunity.

***B. Shutdown- or low-power-based energy saving scenarios***

| **Study (year)** | **Setting / strategy** | **Evidence type** | **Reported or estimated saving** | **Interpretive context** |
| --- | --- | --- | --- | --- |
| Brown et al. (2023) [8] | CT; overnight/weekend system shutdown | Measured + annualised estimate | 14,181 kWh/year | Single-CT after-hours shutdown intervention. |
| Marks et al. (2025) [9] | CT; overnight/weekend shutdown | Measured + annualised estimate | ~140 kWh/week; 7,280 kWh/year | Single-CT shutdown when not clinically required. |
| Stortz et al. (2025) [18] | Regional CT fleet; low-power mode during non-operational hours | Modelling study | 10,717–28,206 kWh/scanner/year | Modelled by scanner utilisation category. |
| Present study (2026) | RT simulation CT; after-hours/weekend shutdown + daytime power-saving | Hypothetical cross-study-aligned estimate | 229.6 kWh/week; 11,939 kWh/year | Scenario-based estimate. |

Note. The present-study value is intentionally reconstructed as a hypothetical cross-study comparison and is not the directly measured daytime intervention effect. To align the scenario with prior shutdown studies, the estimate deliberately includes the contribution of after-hours and weekend shutdown in addition to daytime power-saving, assuming that nights and weekends would otherwise remain powered at baseline idle levels. Consequently, the annualised value should not be interpreted as the energy saving attributable to daytime Power Save Mode alone.
