# Supplementary material for: Daytime Idle Energy Reduction in Radiotherapy Simulation Computed Tomography: A Prospective Workflow‐Based Evaluation
Source: J Med Radiat Sci. 2026 Nov 2:10.1002/jmrs.70133. Online ahead of print. doi: 10.1002/jmrs.70133 (PMC13725422; doi:10.1002/jmrs.70133)
Supplement: Supplementary file 5 [file JMRS-9999-0-s005.docx]

Supplementary Table S3. Extended monitoring during the power-saving period using the revised operational-state definitions

| **Operational state** | **Definition (kW)** | **Time (h)** | **Time (%)** | **Energy (kWh)** | **Energy (%)** |
| --- | --- | --- | --- | --- | --- |
| Standby/non-operational | <1.80 | 15.48 | 7.74 | 23.53 | 3.31 |
| Lower-power state | 1.80–<3.125 | 79.00 | 39.50 | 231.14 | 32.47 |
| Normal operational idle | 3.125–<5.35 | 94.61 | 47.31 | 338.58 | 47.57 |
| Acquisition/high-load | ≥5.35 | 10.91 | 5.46 | 118.58 | 16.66 |

Note. The extended monitoring window was 8 June–13 July 2026. A known ~161-h gap (13 June 07:00 to 20 June 00:00) was not imputed. This table is restricted to available weekday clinical hours (08:00–18:00; 200.0 h). The lower-power state occupied 39.50% of these hours, closely matching the 39.13% observed during the primary 5-day power-saving period. Mean and median power values are intentionally not presented because 5-s averaging can attenuate brief high-power peaks during acquisition and could therefore be misinterpreted as representative acquisition output. The extended analysis is descriptive and is not treated as a second causal estimate of intervention effect.
